# The Effect of Intravenous Autologous Activated Platelet-Rich Plasma Therapy on “Profibrotic Cytokine” IL-1*β* Levels in Severe and Critical COVID-19 Patients: A Preliminary Study

**DOI:** 10.1155/2021/9427978

**Published:** 2021-07-08

**Authors:** Karina Karina, Louis Martin Christoffel, Rita Novariani, Imam Rosadi, Iis Rosliana, Siti Rosidah, Siti Sobariah, Novy Fatkhurohman, Nurlaela Puspitaningrum, Yuli Hertati, Irsyah Afini, Difky Ernanda, Tias Widyastuti, A. D. Sulaeha, Alfida Zakiyah, Noor Aini, Grady Krisandi, Hubert Andrew

**Affiliations:** ^1^Klinik Hayandra, Yayasan Hayandra Peduli, Jl. Kramat VI No. 11, Jakarta, Indonesia; ^2^HayandraLab, Yayasan Hayandra Peduli, Jl. Kramat VI No. 11, Jakarta, Indonesia; ^3^Universitas Pembangunan Nasional Veteran Jakarta, Jakarta, Indonesia; ^4^Pusat Kajian Stem Cell, Universitas Pembangunan Nasional Veteran Jakarta, Jakarta, Indonesia; ^5^Koja Regional Public Hospital, Jl. Deli No. 4, Jakarta, Indonesia; ^6^Department of Biology, Faculty of Mathematics and Natural Sciences, Mulawarman University, Samarinda, Indonesia; ^7^Universitas Indonesia, Jl. Salemba Raya No. 6, Jakarta, Indonesia

## Abstract

**Introduction:**

Elevated concentration of proinflammatory cytokines followed by hyperinflammation is one of the hallmarks of severe and critical COVID-19. In the short term, this may result in ARDS and lung injury; subsequently, this may cause pulmonary fibrosis—a disease with poor prognosis—in the long run. Among the cytokines, interleukin-1*β* (IL-1*β*) is one of the most overexpressed in COVID-19. We speculate that administration of intravenous activated autologous platelet-rich plasma (aaPRP), which contains interleukin-1 receptor antagonist (IL-1RA), would lower IL-1*β* levels and benefit the severe and critical COVID-19 patients.

**Methods:**

After acquiring ethical clearance, we recruited 12 adult COVID-19 patients of both sexes from the Koja Regional Hospital (Jakarta, Indonesia) ICU. After selection, seven patients were included and divided into two groups, severe and critical. In addition to three doses of aaPRP, both groups received the same treatment of antiviral, steroid, and antibiotics. Quantification of plasma IL-1*β* levels was performed by beads multiplex assay a day before the first aaPRP administration and a day after the second and third aaPRP administration. PaO_2_/FiO_2_ ratio and lung injury scores were evaluated a day before and a day after each aaPRP administration.

**Results:**

Severe and critical patients' initial plasma IL-1*β* concentration was 4.71 pg/mL and 3.095 pg/mL, respectively. After 2 treatments with aaPRP, severe patients' plasma IL-1*β* concentration decreased 12.48 pg/mL, while critical patients' plasma IL-1*β* concentration increased to 18.77 pg/mL. Furthermore, after 3 aaPRP treatments, significant amelioration of patients' PaO_2_/FiO_2_ ratio from 71.33 mmHg at baseline to 144.97 mmHg was observed (*p* < 0.05). However, no significant improvement in lung injury score was observed in severe and critical groups. All severe patients and one critical patient recovered.

**Conclusion:**

The use of aaPRP may prevent pulmonary fibrosis in severe COVID-19 patients through the reduction of patients' plasma IL-1*β* concentration and the amelioration of PaO_2_/FiO_2_ ratio.

## 1. Introduction

COVID-19 causes mortality through the process of cytokine storm, which is hyperinflammation and multiorgan damage triggered by uncontrolled release of cytokines. [1] The elevated concentration of proinflammatory cytokines is associated with severe COVID-19 and may result in complications such as acute respiratory distress (ARDS), sepsis, and respiratory failure [1–3]. Such cytokine storms can cause acute lung injury, which, in turn, may induce pulmonary fibrosis [4, 5]. It is estimated that over a third of recovered patients develop fibrotic abnormalities upon radiological examination. As more and more patients recover, post-COVID-19 pulmonary fibrosis is already starting to be reported [6].

Pulmonary fibrosis is a form of chronic, progressive, fibrosing interstitial pneumonia. It is irreversible, has an unpredictable clinical course, and is associated with extremely poor prognosis [7]. As its prevalence is already increasing from other causes [8], additional cases from COVID-19 would give rise to a remarkable morbidity and mortality rate as repercussion. Currently, no definitive cure exists for the condition except for lung transplantation. [9].

Among the overexpressed cytokine present in cytokine storms is interleukin-1 beta (IL-1*β*), which is also implied in the pathogenesis of pulmonary fibrosis [10]. Activated autologous platelet-rich plasma (aaPRP) is a prospective therapy to be performed in COVID-19 patients to combat such systemic inflammation. Apart from its accessibility, it also possesses anti-inflammatory properties through cytokines such as interleukin-1 receptor antagonist (IL-1RA) and a known safety profile [11–14]. Similarly, anakinra, a recombinant IL1-RA, showed promising results in a cohort consisting of patients with severe forms COVID-19 [15].

To our knowledge, no research on the influence of aaPRP administration on cytokine profile in COVID-19 patients has been published previously. Hence, in this study, we explored the effect of intravenous (IV) aaPRP administration on the plasma levels of IL-1*β*-a profibrotic and proinflammatory cytokine in severe and critical COVID-19 patients.

## 2. Methods

This interventional preliminary study recruited 12 patients of both sexes between the age of 18–65. The study protocol was ethically approved by the Health Research Ethics Committee, University of Indonesia and Cipto Mangunkusumo Hospital (HREC-FMUI/CMH), and is registered on ClinicalTrials.gov (NCT04715360).

The inclusion criterion for the study was severe or critical COVID-19 patient being treated in the Koja Regional Public Hospital (Jakarta, Indonesia) ICU. Meanwhile, we excluded patients who were diagnosed with HIV, hepatitis, cancer, chronic kidney disease with ongoing dialysis, and pregnancy. Ultimately, we included seven patients, with three severe and four critical patients enrolled in the study. Critical patients differ from severe patients by the additional employment of mechanical ventilation and administration of vasopressors due to sepsis.

Both groups received Avigan (favipiravir) 1.600 mg twice daily (b.i.d) for a day followed by 600 mg b.i.d. for five consecutive days and oral or parenteral administration of dexamethasone 6 mg/24 hours for 10 days. Oxygen therapy and antibiotics were given accordingly. The patients also received intravenous (IV) aaPRP on day 1, 3, and 5, while quantification of plasma IL-1*β* levels was conducted by the FMUI Integrated Laboratory on day 0, 4, and 6 with PaO_2_/FiO_2_ ratio and lung injury scores recorded with the same intervals. The quantification method was MILLIPLEX^®^ (HCTYTA-60K) beads multiplex assay (Merck, Burlington, Massachusetts), and results were read with the Luminex 200 and Luminex xPONENT (Luminex, Austin, Texas).

aaPRP was prepared using the method invented and developed by HayandraLab. Venous whole blood was collected from each patient into eight sodium citrate tubes and centrifuged at 1.000 rpm for 10 minutes. Blood plasma was then separated and centrifuged at 3.000 rpm for another 10 minutes until platelet sediment formed. The platelet-poor part of the plasma was discarded, and the subsequent plasma was reckoned as inactivated PRP. Activation was achieved by the addition of a calcium activator (H-Remedy, HayandraLab, Indonesia), and the consequent clots were then removed. Finally, the aaPRP was suspended in normal saline and administered intravenously to the patients.

## 3. Statistical Analysis

The paired *T*-test was used to analyze severe, recovered, and dead patients' plasma IL-1*β* profile, and the Friedman test was used to analyze critical patients' plasma IL-1*β* profile. Plasma IL-1*β* profile was further analyzed to compare the profile between the severe-critical group and recovered-dead patients using the Mann–Whitney *U* test. The patients' PaO_2_/FiO_2_ ratio was analyzed using the Friedman test. To compare the PaO_2_/FiO_2_ ratio in the severe and critical group, the Mann–Whitney test and independent sample *T*-test were used. The patients' lung injury score was analyzed using the Friedman test. For comparison of lung injury score in the severe and critical group, the Mann–Whitney *U* test and independent sample *T*-test were used.

## 4. Results

### 4.1. Demography of Patients and Chest X-Ray Finding

A total of 7 patients participated in this study with 3 severe patients (median age: 41 years, 34–55 years) and 4 critical patients (median age: 58 years, 48–61 years). The median age of all patients was 55 years. The youngest patient was 34 years old, and the oldest was 61 years old. All the patients had ARDS and underwent chest x-ray examination with 3 severe patients and 1 critical patient having bilateral pneumonia and 3 critical patients having bilateral opacity. Among the 4 patients who had bilateral pneumonia, 1 critical patient had extensive bilateral pneumonia. All the patients also had underlying comorbidities with some patients having more than 1 underlying comorbidity. The most common underlying comorbidity was type II diabetes mellitus, followed by hypertension, congestive heart failure, obesity, and acute kidney injury. The summary of patients' demography is shown in Table 1.

### 4.2. Patients' Plasma Interleukin-1*β* Concentration and Outcome after Activated Autologous Platelet-Rich Plasma Administration

**Figure 1:**
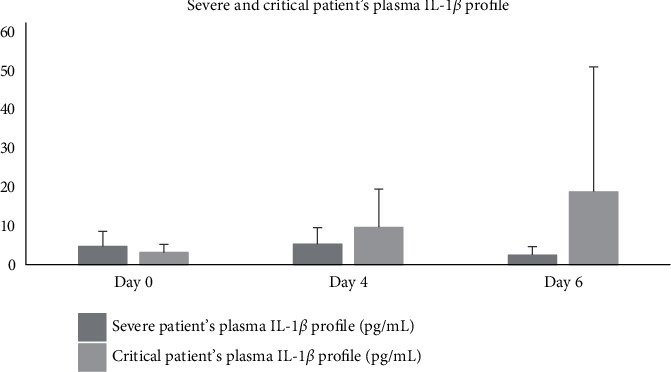
Severe and critical patients' plasma IL-1*β* profile.

**Figure 2:**
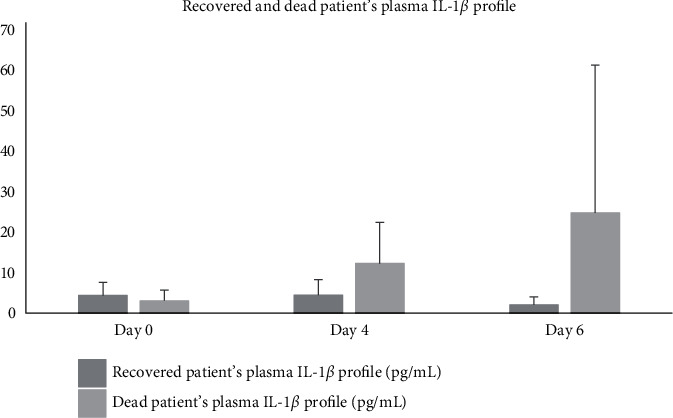
Recovered and dead patients' plasma IL-1*β* profile.

Patients' plasma IL-1*β* profile after aaPRP administration differed between severe and critical patients. Severe patients who underwent aaPRP treatment had a trend of decrease in plasma IL-1*β* concentration from 4.71 pg/mL to 2.48 pg/mL, while critical patients who underwent aaPRP treatment had their plasma IL-1*β* increased from 3.095 pg/mL to 18.77 pg/mL Figure 1. However, both trends were not statistically significant (*p* > 0.05). Furthermore, no statistical significance was observed between the 2 groups (*p* > 0.05). Among the 7 patients who were included in this study, all severe patients and 1 critical patient recovered, while 3 other critical patients died in this study. Patients who recovered had a decreasing trend in plasma IL-1*β* concentration by day 6 of the study, while patients who died had their plasma IL-1*β* concentration increased by day 4 and 6 Figure 2. However, these findings were not statistically significant (*p* > 0.05). Moreover, no statistical significance was observed between the 2 groups (*p* > 0.05).

### 4.3. Patients' PaO_2_/FiO_2_ (PF) Ratio after Activated Autologous Platelet-Rich Plasma Administration

**Figure 3:**
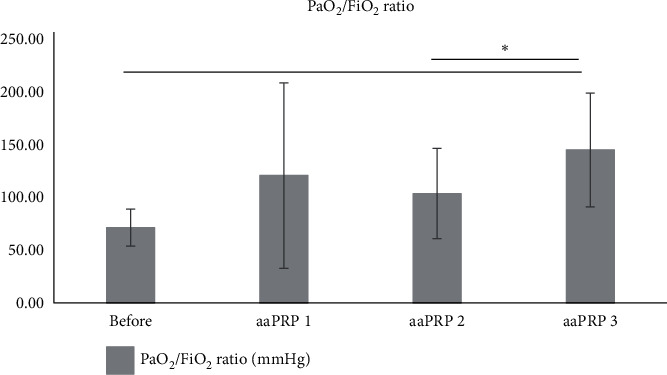
Patients' PaO_2_/FiO_2_ ratio after aaPRP administration.

Patients' P/F ratio was ameliorated after 3 aaPRP administration. A statistically significant increase was observed between before aaPRP administration and after 3 aaPRP administration from 71.33 mmHg to 144.97 mmHg (*p* < 0.05). Furthermore, a statistically significant increase was also observed between the 2^nd^ and 3^rd^ aaPRP administration from 103.7 mmHg to 144.97 mmHg (*p* < 0.05). However, no statistical significance was observed between the severe and critical group (*p* > 0.05), Figure 3.

### 4.4. Patients' Lung Injury Score after Activated Autologous Platelet-Rich Plasma Administration

**Figure 4:**
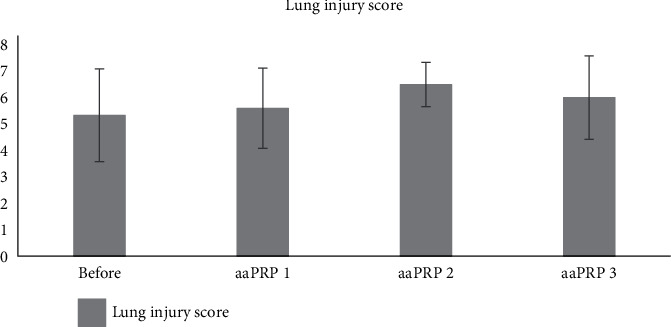
Patients' lung injury score after aaPRP administration.

Patients' average lung injury score increased after 2 aaPRP administration from 5.33 to 6.50 and decreased to 6.00 after the 3^rd^ aaPRP administration. However, the changes of lung injury score after aaPRP administrations were not statistically significant. Moreover, no statistical significance was observed between the severe and critical group (*p* > 0.05) (Figure 4).

## 5. Discussion

Currently, favipiravir is the choice antiviral medication for the treatment of severe and critical COVID-19 patients in Indonesia. Along with favipiravir, dexamethasone is also recommended in for severe and critical patients requiring breathing aid such as oxygen therapy and ventilator [16]. Immunosuppressive therapy along with invasive device usage (such as endotracheal tube for mechanical ventilation) contributes to nosocomial bacterial and fungal infection risk in COVID-19 patients treated in the intensive-care unit (ICU) [17]. Infections may appear as early as one week of ICU stay and significantly extend ICU hospitalization duration [18]; meropenem is given to treat such infection.

Oxygen therapy is initiated if patient peripheral oxygen saturation (SpO_2_) falls below 93% with a nasal cannula (NC) escalating to a nonrebreather mask (NRM); the target SpO_2_ for such therapy is 92–96%. In the event that patient SpO_2_ does not improve after 1 hour of initial oxygen therapy, it is indicated to switch to a high-flow nasal cannula (HFNC). Reevaluation is then performed after two hours to decide whether the patient needs invasive ventilation or not [16]. The maximum achievable fraction of inspired oxygen (FiO_2_) depends on the device used to deliver the oxygen, Table 2. In our ICU, the preferred oxygen therapy is NRM, followed by HFNC and intubation as the last resort. The weaning process was performed by deescalating from a ventilator to an NRM, simple mask, and then, NC.

Huang et al. noted that IL-1*β*, which during cytokine storms is among the most important cytokines in the IL-1 family, is markedly increased in COVID-19 patients [2, 19]. Infection by SARS-CoV-2 induces the release of a proinflammatory cytokine such as IL-1*β* through activation of TLR2 (toll-like receptor 2), TLR3, or TLR4 [20]. IL-1*β* plays an important role in the pathogenesis of pulmonary fibrosis by inducing overexpression of TGF-*β*1, which, in turn, causes a progression to fibrosis via fibroblast differentiation and activation into myofibroblasts [10, 21]. The myofibroblasts cause excessive ECM accumulation which ultimately leads to loss of alveolar function [10].

Blockage of IL-1*β* for COVID-19 is studied in numerous studies. Canakinumab is a fully human IgG monoclonal antibody which targets IL-1*β*. Previously, the efficacy of canakinumab for treatment of COVID-19 was evaluated in CANASCOV, an observational cohort-prospective study; the study progressed to a phase III clinical trial (CAN-COVID) but was suspended due to unsatisfactory interim analysis results [22, 23]. Another IL-1 receptor antagonist, anakinra, was also explored as treatment for COVID-19 in several studies. However, evidence to support anakinra use is still deemed insufficient [24].

Our study aimed at reducing IL-1*β* levels as well as its effects in the body. As mentioned before, aaPRP contains IL-1RA, the endogenous antagonist to IL-1 receptors [11, 12]. It is thought that blockage of IL-1 receptors would reduce inflammation and also fibrosis progression in the long run. While both groups received aaPRP treatment in this study, all the severe patients recovered while only one out of four critical patients survived. It is also observed that IL-1*β* levels are inversely correlated with favorability of patient outcome. Utilization of this relationship may be a valuable predictive biomarker for mortality in COVID-19. However, as all these findings are not statistically significant, it should be interpreted wisely.

We noted the initial increase and subsequent decrease in IL-1*β* levels in patients who recover, while those who died had IL-1*β* levels that continued to increase throughout the study. We speculated that the immunomodulatory effects of aaPRP can prevent sepsis in severe patients [25]. However, in those who are classified as critical and already suffering from sepsis, aaPRP was not able to lower IL-1*β* concentration. Viral septic shock is cited as one of the most notorious cause for death in COVID-19 patients [26]. The initial increase may be a sign of a septic shock that was averted by administration of aaPRP, with the delay in IL-1*β* decrease showing that aaPRP needs time before exerting therapeutic effects. As these findings are unconfirmed, future studies should verify these results.

The therapeutic effect of PRP on lung injury has been studied before *in vivo* by Mammoto et al. [27]. They found PRP to contain various types of angiogenic factors such as angiopoietin-1 and VEGF, which is required for physiological angiogenesis—as opposed to a single angiogenic factor. These angiogenic factors are thought to be the rationale of the vascular and alveolar regeneration observed in the study. Nebulized PRP is also studied for its effects in COVID-19 *in vitro*. Beitia et al. [28] hypothesized that the administration of PRP results in the reduction of fibrosis and the regeneration of damaged pulmonary tissue.

We noted statistically significant improvements in PaO_2_/FiO_2_ (P/F) ratio in our patients over the course of the treatment. Although, it should be noted that, during the duration of the study, none of the patients' PaO_2_/FiO_2_ increased above 300 mmHg. This means all seven patients were still experiencing acute respiratory distress syndrome (ARDS) after the third dose of aaPRP. This result is consistent with the patients' lung injury score which was still above 2.5 after the final dose of aaPRP, indicating the presence of ARDS.

Several modalities have been used to treat severe and critical COVID-19 patients. Among the available modalities, the use of mesenchymal stem cells (MSCs) has been popular ever since due to its immunomodulatory properties [29]. The immunomodulatory properties of MSCs are also known to reduce concentration of fibrotic factors and prevent pulmonary fibrosis in COVID-19 patients. However, the use of MSCs for COVID-19 patients is usually not feasible. The first reason is because collection of autologous MSCs from the patient's own body is not likely to be performed. Furthermore, severe and critical COVID-19 patients are usually present with inflammatory-related comorbidities, such as diabetes mellitus, which reduces the ability of MSCs to expand [30]. Thus, the reduced ability of MSCs to expand will delay the time for more than 2 weeks until it reaches the required dose which will also delay the treatment for severe and critical COVID-19 patients. [31].

As for allogenic MSCs, it has limited potency and safety for use in severe and critical COVID-19 patients. First, it requires healthy donors for the source of MSCs. Second, donor screening and cell characterization are required before the use of allogenic MSCs to prevent immunological rejections and infections. Third, it still requires expansion of MSCs which takes around 2 weeks. Lastly, MSCs are no longer found to be immune-privileged by diminishing HLA-DR because the reexpression of HLA-DR in certain conditions, such as inflammation, may induce immune responses, especially in severe and critical COVID-19 patients where inflammation is prominent [32–34].

As the use of autologous and allogenic MSCs to prevent pulmonary fibrosis is not feasible in COVID-19 patients, aaPRP is a more feasible potential alternative to prevent pulmonary fibrosis in severe and COVID-19 patients. This is because aaPRP can easily be isolated and processed from the patient's venous blood which only requires around 2 hours until it is administered intravenously to the patient. Furthermore, aaPRP has been proven to be safe because it is free of platelets, as proven in our previous studies, which does not cause thrombosis [14].

Nevertheless, there are still several limitations in our study. First, our study is a preliminary study that uses aaPRP to prevent pulmonary fibrosis in only 7 COVID-19 patients. Second, we only present the effect of aaPRP on the concentration of IL-1*β* as a profibrotic parameter. Third, no control patients were included in this study to confirm the reduction of IL-1*β* by aaPRP. Therefore, a bigger sample study on the use of aaPRP to prevent pulmonary fibrosis in severe and critical COVID-19 patients with control and long-term follow-up is required.

## 6. Conclusions

The use of aaPRP in severe COVID-19 patients is found to reduce their plasma IL-1*β* concentration which is beneficial to prevent pulmonary fibrosis after they have recovered. As for critical COVID-19 patients, their plasma IL-1*β* concentration increased but one of the patients recovered from COVID-19. Furthermore, patients' PaO_2_/FiO_2_ ratio improved significantly indicating improvement of lung function.

## Figures and Tables

**Table 1:** Demography of patients and chest x-ray findings.

Patient code	Group	Sex	Age	Comorbidities	Chest x-ray	ARDS	Outcome
PRP 1	Severe	F	55	Type II diabetes mellitus and obesity	Bilateral pneumonia	Yes	Recovered
PRP 8		F	34	Hypertension	Bilateral pneumonia		Recovered
PRP 10		M	41	Obesity	Bilateral pneumonia		Recovered

PRP 2	Critical	F	48	Hypertension	Extensive bilateral pneumonia	Yes	Dead
PRP 7		F	56	Type II diabetes mellitus and obesity	Total bilateral opacity		Recovered
PRP 11		F	61	Type II diabetes mellitus, congestive heart failure, and acute kidney injury	6/3 bilateral opacity		Dead
PRP 12		M	60	Type II diabetes mellitus, hypertension, and congestive heart failure	2/3 bilateral opacity		Dead

**Table 2:** Various oxygen delivery devices.

Device	Flow (L/minute)	Achievable FiO_2_
Nasal cannula	3–6	Maximum ∼30%
Simple mask	6–8	Maximum ∼60%
Rebreathing mask	10–12	Maximum ∼80%
Nonrebreather mask	12–15	Maximum ∼95%
High-flow nasal cannula	25–60	40–100%
Intubation	30–40	Any FiO_2_

## Data Availability

The data used to support the findings in this study are included within the article.
