# Corrigendum to “The Effect of Intravenous Autologous Activated Platelet-Rich Plasma Therapy on “Profibrotic Cytokine” IL-1*β* Levels in Severe and Critical COVID-19 Patients: A Preliminary Study”

**DOI:** 10.1155/2023/9846961

**Published:** 2023-02-03

**Authors:** Karina Karina, Louis Martin Christoffel, Rita Novariani, Imam Rosadi, Iis Rosliana, Siti Rosidah, Siti Sobariah, Novy Fatkhurohman, Nurlaela Puspitaningrum, Yuli Hertati, Irsyah Afini, Difky Ernanda, Tias Widyastuti, A. D. Sulaeha, Alfida Zakiyah, Noor Aini, Grady Krisandi, Hubert Andrew

**Affiliations:** ^1^Klinik Hayandra, Yayasan Hayandra Peduli, Jl. Kramat VI No. 11, Jakarta, Indonesia; ^2^HayandraLab, Yayasan Hayandra Peduli, Jl. Kramat VI No. 11, Jakarta, Indonesia; ^3^Universitas Pembangunan Nasional Veteran Jakarta, Jakarta, Indonesia; ^4^Pusat Kajian Stem Cell, Universitas Pembangunan Nasional Veteran Jakarta, Jakarta, Indonesia; ^5^Koja Regional Public Hospital, Jl. Deli No. 4, Jakarta, Indonesia; ^6^Department of Biology, Faculty of Mathematics and Natural Sciences, Mulawarman University, Samarinda, Indonesia; ^7^Universitas Indonesia, Jl. Salemba Raya No. 6, Jakarta, Indonesia

In the article “The Effect of Intravenous Autologous Activated Platelet-Rich Plasma Therapy on “Profibrotic Cytokine” IL-1*β* Levels in Severe and Critical COVID-19 Patients: A Preliminary Study” [1], the authors would like to update the Methods section as follows.

## 2. Methods

This interventional preliminary study recruited 12 patients of both sexes between the age of 18–65 years as interim data of Phase II clinical trials. The study protocol was ethically approved by the Health Research Ethics Committee, University of Indonesia, and Cipto Mangunkusumo Hospital (HREC-FMUI/CMH) and is registered on ClinicalTrials.gov (NCT04715360).
